# The Effects of Voxel Localization and Time of Echo on the Diagnostic Accuracy of Cystic Brain Tumors in 3 Tesla Magnetic Resonance Spectroscopy

**DOI:** 10.5812/iranjradiol.7510

**Published:** 2012-11-20

**Authors:** Alireza Rezvanizadeh, Kavous Firouznia, Mohammad Salehi-Sadaghiani, Meisam Mohseni, Dona Gharaei, Hossein Ghanaati, Hamidreza Saligheh Rad, Majid Masoudnia

**Affiliations:** 1 Department of Radiology, Faculty of Medicine, Tehran University of Medical Sciences, Tehran, Iran; 2 Advanced Diagnostic and Interventional Radiology Research Center (ADIR), Tehran University of Medical Sciences, Tehran, Iran; 3 Faculty of Medicine, Tehran University of Medical Sciences, Tehran, Iran; 4 Department of Neurosurgery, Faculty of Medicine, Tehran University of Medical Sciences, Tehran, Iran; 5 Department of Medical Physics and Biomedical Engineering, Faculty of Medicine, Tehran University of Medical Sciences, Tehran, Iran

**Keywords:** Brain Neoplasms, Choline, Creatinine, Magnetic Resonance Spectroscopy, N-acetylaspartate, Cystic

## Abstract

**Background:**

Although magnetic resonance spectroscopy (MRS) has been shown as an effective diagnostic tool in distinguishing inflammation from neoplasm in cystic brain lesions, the optimum approach in selecting the portions of lesions in MRS and the possible effects of different times of echoes (TEs) remains unknown.

**Objectives:**

To determine the most effective TE in diagnosing neoplastic lesions based on detecting choline (Cho), N acetyl aspartate (NAA) and creatinine (Cr). Moreover, the role of voxel localization on the diagnosis of the neoplastic nature of the lesions is assessed through comparing the abovementioned metabolite ratios in the rim and center of each lesion with the same TE.

**Patients and Methods:**

In 16 patients with brain cystic tumors, MRS was performed at TEs of 30, 135 and 270 ms for detection of Cho, NAA and Cr metabolites using a 3 tesla MRI unit. The percentage of analyzed ratios greater than a cut-off point of 1.3 for Cho/Cr and 1.6 for Cho/NAA were calculated.

**Results:**

Cho/Cr and Cho/NAA ratio means at all TEs were more at the central area in comparison with the periphery, although none of the differences were statistically significant. There was no statistically significant difference among the compared TEs. The percentages of ratios above the cut-off point at all TEs were more in the rim compared to the center and in the union of both compared to the rim or center. All the patients had at least one voxel with a Cho/Cr ratio of more than 1.3 when the voxel was chosen according to the hotspots shown in the chemical shift imaging map, regardless of their location at all examined TEs.

**Conclusions:**

Selection of voxels with the guide of chemical shift imaging map yields to 100% diagnostic sensitivity. If not accessible, the use of the union of peripheral and central voxels enhances the sensitivity when compared to usage of peripheral or central voxels solely.

## 1. Background

Brain tumors comprise a diverse group of neoplasms. Despite the fact that brain tumors only comprise 2 percent of all cancers, they lead to a disproportionate morbidity and mortality rate (1). Historically, in the past decades, many imaging modalities have been invented to differentiate brain tumors from other mass lesions (2, 3). The differential diagnoses usually have different nature, prognosis and treatment. Moreover, the differential diagnoses grow wider and more diverse when they have central cystic or necrotic portions, such as abcess, resolving hematoma, radiation necrosis and masses with developmental origin (4, 5). It is shown that conventional MRI has only 61.4% sensitivity in differentiating brain cystic neoplasms from abcess as their main differential diagnosis (6). Newer imaging methods such as diffusion weighted imaging (DWI) and proton magnetic resonance spectroscopy (MRS) were shown to light the blind spots of conventional MRI with significant increase in diagnostic accuracy (5, 6) when used as an adjunct. MRS is a non-invasive diagnostic technique which can determine the concentration of specific metabolites in a pre-selected volume of tissue. These modalities have been widely used to assess the pathophysiology of different neurological abnormalities, e.g. epilepsy (7) and Parkinson’s disease (8). In recent years, MRS is finding a role in practice, as a promising diagnostic technique in detecting various neurological and neurosurgical disorders, including focal brain lesions (9). It is now widely accepted that MRS can distinguish tumors and non-neoplastic lesions based on the Choline (Cho) to Creatinine (Cr) ratio (10). Further, some studies showed the correlation between the tumor grade and Cho/Cr or Cho to N-Acetyl Aspartate (NAA) values (11-14) and some articles discussed differentiation of primary infiltrating from metastatic tumors by increased Cho/Cr ratios at peritumoral regions (14), although there is no global consensus on both issues yet. In addition, it has been shown that the enhancing rim area which includes infiltrating tumor cells in glioblastoma multiform or inflammatory cells in pyogenic abcess is useful in distinguishing the lesions (15). However, the possible effectiveness of determining metabolite concentration at the center of the brain lesions with cystic components and its comparison with the rim area remains unknown. This issue shows its worth when a radiologist is faced with several voxels in a multivoxel MRS, some covering the central cystic components and some covering the peripheral regions. Then it is vital to know which voxels are considered more important and weigh more. Some previous studies used peripheral enhancing rim as the preferred site of sampling, while others used the central parts (5, 6, 16). Whilst peripheral enhancing rim sounds to be the viable portion of the tumor with a greater cellular turnover rate, peripheral voxels are more susceptible to signal contamination from the adjacent normal tissue, a fact that can reduce the accuracy of these voxels. Another fact which should be considered is that MRS acquisition at 1.5 tesla MRI units with short time of echo (TE) protocols are not as accurate as long TE ones, if NAA, Cho or Cr are addressed (17). However, there is paucity of data focusing on this issue in 3 tesla imagers, especially in clinical practice.

## 2. Objectives

The answers to the mentioned problems will provide a more optimal approach in MRS usage for the diagnosis of neoplastic tissues. In the current study, the effects of three different TEs (30, 135 and 270) are assessed to determine the most effective TE in diagnosing neoplastic lesions based on detecting Cho, NAA and Cr. Moreover, the role of voxel localization on the diagnosis of the neoplastic nature of the lesions is assessed through comparing the abovementioned metabolite ratios in the rim and center of each lesion with the same TE.

## 3. Patients and Methods

### 3.1. Subjects

Twenty-two patients with brain mass lesions in retrospective MR study were prospectively re-evaluated with conventional MR imaging and MR spectroscopy before undergoing surgery. On conventional MRI, all the patients had lesions with central cystic components with an approximately similar signal to CSF on T1-weighted and T2-weighted images and no central enhancement on post-contrast images. Among these patients, 16 had brain tumors according to the pathologic result and were included in the study. The population included 12 men and 4 women ranging from 1 to 75 years of age (mean age, 41.4 years). Before MR examination, informed consent was obtained from all participants for MR studies and for review of patients’ records and images.

### 3.2. MR Imaging Evaluation

All participants were examined using a 3-T superconducting MR imaging unit (Magnetom Vision Plus; Siemens, Erlangen, Germany) with a standard circularly polarized head coil in the Medical Imaging Center, Imam Khomeini Hospital Complex. In each participant, axial T1-weighted (500/8.5 ms [TR/TE]) spin-echo (SE), T2-weighted (5000/77 ms) turbo SE and fast fluid-attenuated inversion recovery (FLAIR) (9200/93/2522 ms [TR/TE/TI]) images were obtained using 5-mm section thickness, 220-mm field of view (FOV) and 160 × 256 matrix sizes. After intravenous administration of gadopentetate dimeglumine 0.1 mg/kg (Magnevist, Bayer Schering Pharma, Germany), contrast-enhanced T1-weighted SE sequences were obtained in axial, coronal and sagittal planes. In all cases, normal, cystic or necrotic portions and an enhancing tumoral margin, if existed, were defined on the basis of the following imaging features: normal tissue, an area containing no enhancement and normal signal intensity on T2-weighted images selected from the contralateral normal hemisphere; central cystic or necrotic portions, a region containing signal intensity similar to CSF on both T1W and T2W images- except for hemorrhagic cystic changes which may have high signal intensity on T1W images- without contrast enhancement, and finally peripheral enhancing margin, thick nodular or thin ring enhancement with abnormal signal on T2W images around the cystic center.

### 3.3. Proton MR Spectroscopic Imaging

As the literature seems to demonstrate that gadolinium has a negligible effect on metabolite ratios and peak areas (18-20), multivoxel proton chemical shift imaging (CSI) or spectroscopic imaging was performed after administration of gadopentetate dimeglumine. A section with 5-mm thickness was obtained in the axial plane for all patients. The hybrid multivoxel CSI technique uses a point-resolved spectroscopy (PRESS) double spin-echo sequence for preselection of a volume of interest (VOI) that was usually defined to include the abnormality as well as normal-appearing brain tissue when possible. To prevent strong contribution to the spectra from subcutaneous fat signals, the VOI was completely enclosed within the brain and positioned at the center of the phase-encoded field of view, which was large enough to prevent wrap around artifact. Saturation bands were placed adjacent to VOI. A typical VOI consisted of a 7 × 6 cm^2^ region placed within a 16 × 16 cm^2^ field of view on a 1.5 cm transverse section. A 16 × 16 phase-encoding matrix was used to obtain the 6 × 7 array of spectra in the VOI, with an in-plane resolution of 1 × 1 cm^2^ and a voxel size of 1 × 1 × 1.5 cm^3^. The metabolite peaks were assigned as follows: Cho, 3.22 ppm; Cr, 3.02 ppm; and NAA, 2.02 ppm. This protocol was repeated three times for every participant, but every time with a different time of echo (TE): 30,135 and 270 ms. Then a voxel was chosen in the central cystic or necrotic region, peripheral enhancing rim and the contralateral normal hemisphere for any session of the MRS study. Afterwards, Cho, Cr and NAA peak integral amount of chosen voxels were wrote down. The data gathered from the central cystic portion, peripheral enhancing rim and normal side were respectively known with a suffix of -c (standing for central), -r (standing for rim), -n (standing for the normal side). There were no criteria for voxel selection, except exclusion of noisy voxels, contaminated with CSF or skull fat signals.

### 3.4. Statistical Analysis

As it has been mentioned above, depending upon the TE and the location of the voxel, different values of Cho, Cr and NAA peaks were obtained. Then Cho/Cr and Cho/NAA were calculated in different TEs and different locations. These ratios were analyzed in two separate ways: first, different location ratios were compared in any TE to check the effect of voxel location on the ratios; second, the ratios of either the center or the rim were compared to their analogous location in different TEs. The latter analysis was performed to check the effect of TE changes on the ratios. In order to assess the location effect, Wilcoxon signed rank test was used and to study the role of different TEs, Friedman test was used. Cho/Cr ratios less than 1.3 can be found in normal white matter, while greater values are detected in tumoral tissue (16). Similarly, in another study it was shown that NAA/Cho less than 0.61 (or Cho/NAA more than 1.6) (21) has an acceptable sensitivity and specificity to differentiate the tumoral lesion from normal tissue. In this study, we used the cut-off point of 1.3 for Cho/Cr and 1.6 for Cho/NAA for diagnostic values. Thus, to analyze the percentage of ratios more than aforementioned cut-off points, Cochran’s Q test was performed. A P value less than 0.05 was considered statistically significant. To ensure quality control of spectroscopic data, normal values for NAA/Cr and Cho/Cr were obtained in normal-appearing white matter in the contralateral normal hemisphere.

**Figure 1:**
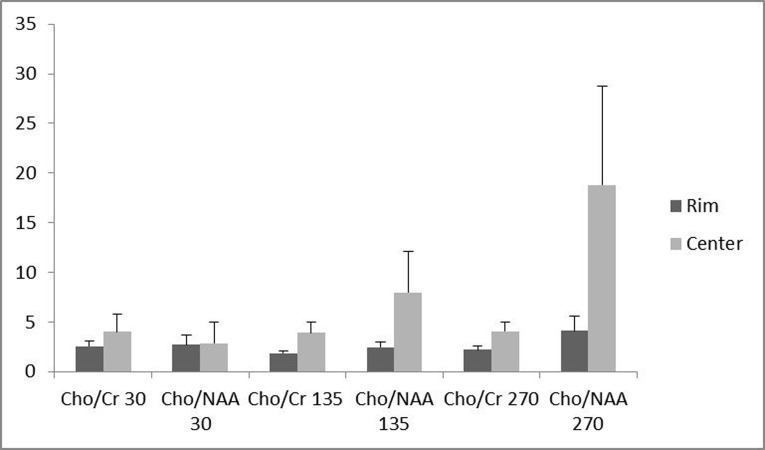
Assessment of voxel localization effect on the analyzed metabolites ratios. The relevant TE number of each ratio is shown at the axis, while the color of each column represents the associated localization of voxels (center vs. rim). The height of bars represents mean.

**Figure 2:**
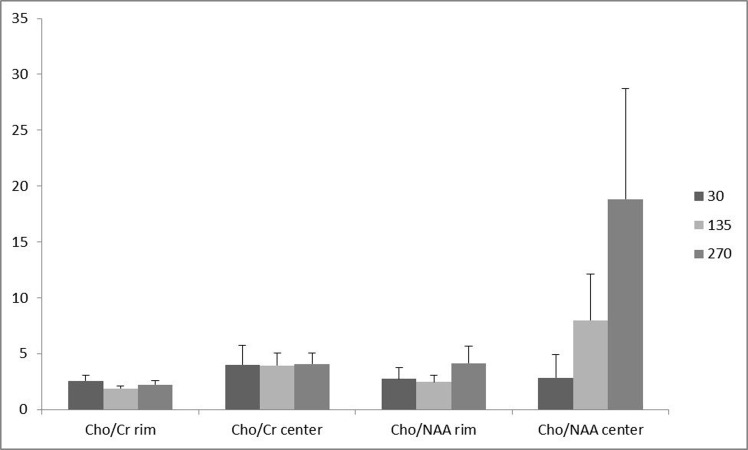
Comparing the effect of different TEs on the analyzed metabolite ratios. Each triple column represents a specific location. The color of ratios corresponds to the TE. The height of bars represents mean.

## 4. Results

Of the 16 patients, 10 (62.5 %) had glioblastoma multiform (WHO grade IV) based on postoperative pathologic specimens reviewed by an experienced neuro-pathologist. One (6.25%) pilocytic astrocytoma, one (6.25%) oligodendroglioma (WHO grade I), two (12.5%) anaplastic astrocytoma (WHO grade III) and two (12.5%) metastases were also reported. The metabolite peak amplitude of Cho, NAA and Cr were analyzed. The Cho/Cr and Cho/NAA ratios for the rim and the center of the cystic tumors were computed separately for TE = 30, 135 and 270 ms (Figure 1). In order to investigate the role of the voxel location on the resulting ratios, the associated ratios of the rim and the center of the tumors were compared. As demonstrated in Figure 1, the Cho/Cr and Cho/NAA ratio means at all TEs were more at the central area, although none of the differences were statistically significant (Figure 1). Furthermore, in order to assess the effect of TE, the abovementioned ratios were compared in different TEs (Figure 2). There was no statistically significant difference among the compared TEs.

**Figure 3:**
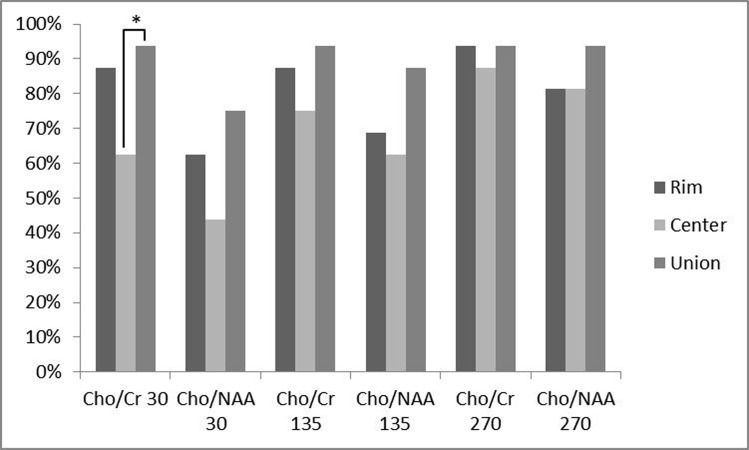
Comparing the percentage of patients with a metabolite ratio higher than a cut-off point of 1.3 for Cho/Cr ratios and 1.6 for Cho/NAA ratios and their relation to the voxel location. Any triple bar group corresponds to a specific ratio-TE which are tagged under the bars. The color of each bar indicates the location of assessment (rim vs. center vs. union of them). Values with * differ significantly (0.01 < P < 0.05).

In addition, the percentage of analyzed ratios greater than a cut-off point of 1.3 for Cho/Cr ratio and 1.6 for Cho/NAA ratio were calculated for all ratios at different TEs and locations (Figure 3). At TE = 30, 87.5% of Cho/Cr ratios at the peripheral area were above the cut-off point, while this percentage decreased to 62.5% at the central portion and the union of these two places resulted in 93.75% above the cut-off point. The percentages greater than the cut-off point, for Cho/NAA ratio at TE = 30 were 62.5% and 43.75% for the rim and center, respectively. Considering the two ratios together, the percentage increased to 75%. At TE = 135, 87.5% of Cho/Cr ratios at the peripheral area and 75% at the central area exceeded the cut-off point. The union of the rim and center leaded to 93.75% of the ratios above the cut-off point. For Cho/NAA ratio at TE = 135, the percentages of ratios which exceeded the cut-off point were 68.75%, 62.5% and 87.5% for the rim, center and their union, respectively. The resultant percentages of Cho/Cr ratios at TE = 270 above the defined threshold for the peripheral and central area and their union were 93.75%, 87.5% and 93.75%, respectively. For Cho/NAA ratio at TE = 270 the calculated percentages of ratios above the cut-off point were 81.25%, 81.25% and 93.75% in the order mentioned previously. In a same trend for all applied TEs, the percentages of ratios above the cut-off point were more in union of the center and the periphery. In addition, the peripheral area showed greater numbers compared with the central ratios. Cochran’s Q test was used to assess the differences between percentages of the ratios above the cut-off point in the rim, center or union of both. As it is depicted in Figure 3, there was a statistically significant difference in the Cho/Cr ratio of the center and union of the central and peripheral area, revealing a statistically significant difference (*P* < 0.05) at TE = 30. Finally, all the patients had at least one voxel with a Cho/Cr ratio of more than 1.3, when the voxel was chosen according to the hotspots shown in the chemical shift imaging map of Cho/Cr ratios, regardless of their location in the center or rim at all examined TEs.

## 5. Discussion

Intracranial cystic mass lesions represent a significant neurosurgical dilemma. Based on nonspecific clinical findings and tumor appearance on imaging modalities including CT scan and conventional MRI, differentiation of various intracranial cystic lesions may be challenging (6). Hence, newer imaging modalities such as multivoxel MRS have been utilized to increase the accuracy of diagnosis. However, up to this time, there has been no study addressing the effect of voxel localization and TE on the diagnostic accuracy of cystic mass lesions. Based upon the importance of the ring enhanced area, some previous studies selected the peripheral voxels to investigate the role of MRS in discriminating neoplastic and non-neoplastic lesions, while other studies have focused on the central portion of brain tumors that is more prone to necrosis (5, 6, 14, 22, 23). Some studies using single voxel MRS selected both the ring enhanced area and non-enhanced inner portion of brain tumors (24). The problem remains an issue in multi-voxel MRS. According to our knowledge; no study has been conducted to investigate the optimal region of the study in brain cystic lesions for MRS diagnosis. It is approved that choline is attributable to trimethyl ammonium residues of free choline as well as phosphocholine, phosphatidylcholine and glycerophosphocholine. This metabolite reflects cell membrane synthesis and degradation. Thus, all processes resulting in hypercellularity (e.g., primary brain neoplasms or gliosis) or myelin breakdown (demyelinating diseases) lead to locally increased Cho concentration. Hence, in theory, the ring enhanced portion of focal cystic brain tumors that contains viable cells with higher rates of turnover sounds as a proper area in discrimination of neoplastic and non-neoplastic lesions. However, according to the results of the current study, the mean value of the aforementioned metabolite ratios (Cho/Cr and Cho/NAA) were greater in central voxels in comparison with peripheral ones at all TEs, although the difference was not significant at any TE. This pattern can probably be explained by two theories. First, contamination of the tumoral tissue Cho signal in the borders, by the adjacent normal parenchymal tissue is a potential fact that might reduce the Cho signals and hence Cho/Cr and Cho/NAA ratios in peripheral voxels. In other words, the voxels with the aforementioned sizes at the rim, usually not only contain a thinner tumoral rim, but also include the normal adjacent tissue. This phenomenon can lead to a decrease in Cho metabolite concentration in the selected voxel area. Although the mentioned contamination can be minimized by decreasing the voxel size, the resultant decrease in signal/noise ratio remains a limitation. This phenomenon is augmented in metastases, where peritumoral abnormal signals are believed to reflect mere vasogenic edema without infiltrating the tumoral cell (25, 26). On the other hand, in the glioma, the peritumoral edema outside the margins of tumoral contrast enhancement contains nests or pseudopods of tumoral cells (27). Thus, metastases are more prone to contamination with normal tissue signals in tumor boundaries. The second potential reason explaining the greater values of Cho/Cr and Cho/NAA ratios in central voxels are the possible effect of gadolinium (Gad)-based contrast on the peripheral enhancing rim. Although some previous studies demonstrated that gadolinium has a negligible effect on metabolite ratios and peak areas (18-20), these studies were performed on 1.5 Tesla units. Unfortunately, the augmented effect of Gad on metabolites, in higher power fields, has not been evaluated yet. However, the results probably show that spatial signal contamination with adjacent tumor tissue and/or Gad effect on Cho signal peak may exert a notable impression on Cho and Cho/Cr ratios, which can overweigh the higher cellularity in the peripheral area.

**Figure 4:**
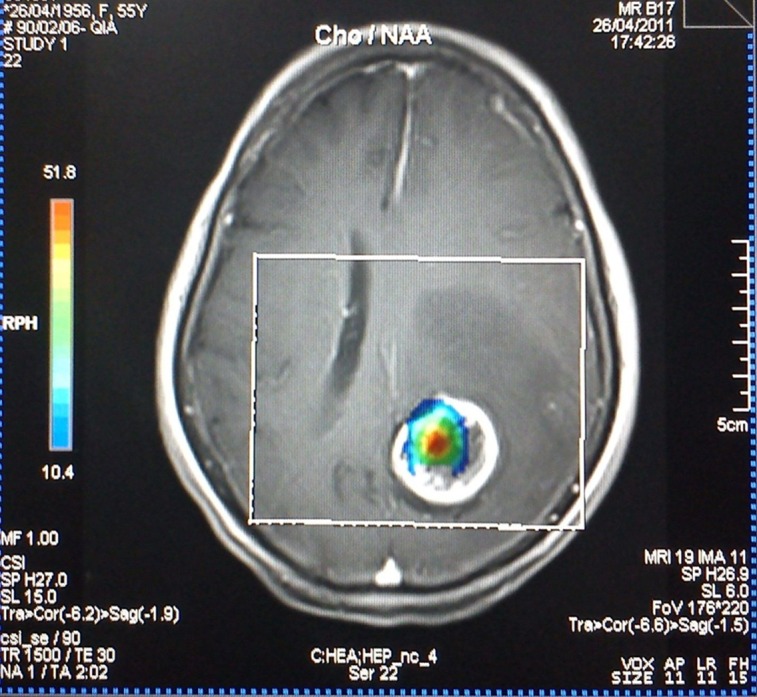
MR spectroscopy of parietooccipital brain lesion on the left hemisphere is depicted along with the hotspot localization. With the guidance of chemical shift imaging map hotspot a more reliable data is gathered.

The effect of voxel localization was also tested in another way. In practice, Cho/Cr and Cho/NAA values above or below an assumptive threshold can be an acceptable indicator of the neoplastic or nonneoplastic nature of a lesion. However, there is no global consensus on a unique threshold and the sensitivity and specificity can vary with the utilized threshold amount. According to previous studies and acceptable reported sensitivity of 1.3 for Cho/Cr and 1.6 for Cho/NAA as a threshold (17, 22), we used the abovementioned thresholds as the diagnostic cut-off point in the present study. On TE = 135, 25% of all the patients showed ratios less than 1.3 in the central regions. While for the peripheral voxels, this number decreased to 12.5%. The union of both the periphery and the center resulted in higher percentages of ratios above the cut-off point (93.75%). Similarly, the percentages of ratios among other TEs, which were above the threshold in the center, rim and union of both locations, showed an increasing manner, although the differences were not significant, except for Cho/Cr in the center (62.5%) and union of the center and the rim (93.75%) at TE = 30. The smaller mean of metabolite ratio values in combination with the greater fraction of ratio values above the threshold in the periphery in comparison with the center, indicates the heterogeneous nature of central voxels. In other words, some of the central voxels have greater metabolite ratio values relative to peripheral voxels (the ones much more than the threshold) that result in a greater mean, while there are many other central voxels with ratio values under the defined threshold. On the other hand, the peripheral voxels show a smaller ratio mean, with possible aforementioned reasons, but with a greater fraction above the defined threshold. In essence, it can be interpreted that the peripheral values show a more homogeneous pattern. We also found that selection of voxels with the guide of chemical shift imaging map hotspots (Figure 4) results in 100% sensitivity in all TEs. Hence, it is wise to select the voxels according to the chemical shift imaging map, instead of restriction to the rim or cystic parts. If not accessible, the best approach not to miss any high Cho/Cr or Cho/NAA ratio is selection of both the rim and center. Furthermore, peripheral voxels showed increased computed ratios in comparison with the central ones, when they are solely assessed, although the observed difference was not statistically significant.

We did not detect any differences among the investigated TEs. The effect of TE on Cho, Cr and NAA were well demonstrated in previous studies on 1.5 tesla imagers. In theory, in longer TEs such as 135 or 270, differences between NAA, Cho and Cr signal intensities are maximized because of the differences in the concentration of these metabolites and T1 relaxation times in tumors versus normal tissues (28). The T1 relaxation time of Cho is longer than that of either Cr or NAA; thus, the ratios of Cho/Cr and Cho/NAA will be proportionally larger at echo times greater than 135 ms compared to echo times less than 30 msec. Furthermore, short TE spectra display greater baseline distortion and estimating signal areas calls for more sophisticated software algorithms processing (17). Notwithstanding the potential role of TE in theory, in practice, the results suggested that probably there is no significant difference between short and long TEs in higher field units of 3 tesla MRI.

However, the use of commercial edition of SYNGO® software, installed on Siemens 3 tesla MRI unit for the analysis of metabolite ratios remained a limitation of our study. Although noisy or distorted baseline voxels were eliminated by the neuroradiologist of the group to overcome the possible incomplete water signal suppression by SYNGO software, we believe that further evaluations with greater number of patients and smaller voxel sizes, performed before and after contrast injection can add valuable information to the current study. Previously, it was shown that in comparison with normal brain tissue, Cho signal reveals more values in tumoral tissues. The optimal place for voxel selection in detecting these changes in spectra has been unspecified. An unanticipated finding in the current study was that Cho/Cr ratio was increased significantly at the center of brain cystic tumors in comparison with the rim. Selection of voxels with the guide of chemical shift imaging map yields to 100% diagnostic sensitivity. If not accessible, the usage of the union of peripheral and central voxels enhances the sensitivity when compared to usage of peripheral or central voxels solely. In addition, increasing TE from 30 to 135 and 270 ms does not have any significant effect on Cho/Cr and Cho/NAA values.
